# Exploring the Socio-Demographic and Psychosocial Factors That Enhance Resilience in the COVID-19 Crisis

**DOI:** 10.3390/ijerph191912580

**Published:** 2022-10-01

**Authors:** Snow Yunni Lin, Jian Han Tan, Brenda Xian Hui Tay, John Paul Chern Shwen Koh, Lei Siew, Marcus Cher Hean Teo, Jeremy Yen Chin Tan, Saima Hilal

**Affiliations:** 1 MBBS Programme, Yong Loo Lin School of Medicine, National University of Singapore, Singapore 117597, Singapore; 2 Saw Swee Hock School of Public Health, National University of Singapore and National University Health System, Singapore 117549, Singapore

**Keywords:** COVID-19, resilience, psychosocial, sociodemographic, Singapore

## Abstract

The Coronavirus disease 2019 (COVID-19) has greatly affected mental health worldwide. This study aimed to identify sociodemographic and psychosocial factors that influence the level of resilience among Singaporeans amidst the pandemic. An online questionnaire was administered to Singaporeans and permanent residents aged 21 and above. The online questionnaire collected information on sociodemographics, infection, and contact with COVID-19. Psychosocial variables—specifically optimism, self-efficacy, hope, and resilience—were also assessed through validated questionnaires. A total of 404 responses were collected in this study. Men were reported to have higher resilience compared to women (28.13 vs. 25.54, *p*-value < 0.001). Married individuals were observed to have higher resilience compared to their single counterparts (27.92 vs. 25.77, *p*-value < 0.001). Interestingly, participants who knew of family members/friends who had contracted COVID-19 were reported to be more resilient than those who did not (28.09 vs. 26.19, *p*-value = 0.013). Optimism, self-efficacy, and hope were also found to be associated with higher resilience (*p*-value < 0.001). In conclusion, one’s sex, marital status, contact with COVID-19, level of optimism, self-efficacy, and hope were shown to significantly affect resilience. Given the long-drawn nature of the COVID-19 pandemic, interventions should aim to improve optimism, self-efficacy, and hopefulness in the community.

## 1. Introduction

The Coronavirus disease 2019 (COVID-19) began as viral pneumonia in China in late 2019 and, within a few months, took over the entire world as a pandemic [1]. The safety measures taken against this pandemic have included social distancing measures, quarantine, and travel lockdowns, which have now become the ‘new normal’. These actions have tremendously changed the routine life of the general population. They have also led to detrimental psychological effects, such as depression, low mood, irritability, and post-traumatic stress—some of which may be long-lasting, resulting in a mental health crisis [2]. Given the detrimental effects of COVID-19 on mental health, there is increasing emphasis on the importance of enhancing resilience amongst individuals to alleviate the adverse outcomes of mental stressors precipitated by the pandemic.

Resilience is commonly defined as the measure of successful stress-coping ability or the embodiment of personal qualities enabling one to thrive in the face of adversity [3]. Resilience can be understood as an outcome in which there is an absence of psychopathological symptoms or as a more complex process involving the individual’s adaptive (cognitive, emotional, and behavioral) reactions [4,5,6]. Resilience, being a multi-dimensional characteristic, is influenced by one’s life experiences, context, time, age, gender, and cultural origin [3]. With internal and external stressors being ever-present in our lives, one’s ability to cope is determined by both successful and unsuccessful adaptations. In some situations, these adaptations are ineffective, resulting in the disruption of biopsychospiritual homeostasis, while others are reintegrative, representing an opportunity for growth and increased resilience [3]. Due to the importance of flexibility in resilient outcomes, it is essential to assess those specific protective factors that promote high levels of resilience in situations caused by the COVID-19 pandemic [7]. Given the ever-changing social distancing policies due to COVID-19, there is increased pressure on the public to adapt swiftly to the ‘new normal’ to prevent any further spread of the virus [8]. Hence, resilience is key in facilitating this process, ensuring that individuals have the psychological capacity to deal with negative events and continue to adapt and recover [9].

A recent study showed that key sociodemographic factors, such as social position and household affordances, are significant predictors of lockdown coping and resilience [10]. Other studies reported that age, education level, and occupation influence one’s resilience during the pandemic [11]. Aside from social demographic factors, psychosocial factors, such as being hopeful and having increased self-efficacy, are associated with better life satisfaction and lower anxiety, thereby improving one’s resilience and post-traumatic growth against the stresses caused by COVID-19 [12,13,14].

Like the rest of the world, Singapore has also reported overwhelming cases of COVID-19 infections. Several welfare organizations have observed a spike in distress calls to their helplines since the start of the pandemic. Samaritans of Singapore (SOS), a non-profit organization focusing on crisis intervention and suicide prevention, revealed a 22% increase in calls to their 24 h hotline in 2020 compared to 2019, further emphasizing the need to study resilience in the local community [15]. Therefore, this study aimed to identify the sociodemographic and psychosocial factors that significantly influence the level of resilience among Singaporeans amidst the COVID-19 pandemic, particularly those that may act as protective factors.

## 2. Materials and Methods

### 2.1. Study Design

This cross-sectional study employed the non-probability sampling methods of voluntary, snowball, and convenience sampling, which were achieved through the distribution of an online questionnaire using the following methods: social media posts on Instagram; messages shared on other communication platforms, such as WhatsApp and Telegram; and assistance from internal organizations, such as the National University of Singapore (NUS) Medical Society. Details regarding the study were included in the questionnaire to ensure informed consent. Consent was obtained in the form of written consent, as participants were required to tick a box stating that they consented to the study before proceeding with the questionnaire.

The online questionnaire was hosted through REDCap from 4 October to 17 October 2021, after the commencement of the stabilization phase of the transition to COVID-19 resilience measures on 27 September 2021. To increase survey outreach among the Singapore population, the social media posts, messages, and questionnaires utilized were made available in four of Singapore’s national languages: English, Chinese, Malay, and Tamil. Participants were informed of the study’s objectives, the voluntary nature of participation, and the omission of personal identifiers, such as IP addresses.

Overall, 485 participants attempted the questionnaire—of which, 80 responses were incomplete with an additional one participant who did not meet the age criteria specified—leaving a total of 404 valid responses (83.3% completion rate). Ethics approval was sought from the Departmental Ethics Review Committee (DERC), NUS (reference number: SSHSPH-148).

### 2.2. Inclusion and Exclusion Criteria

The inclusion criteria were: (1) Singapore Citizens (SCs) or permanent residents (PRr) above the age of 21, and (2) individuals that were able to read and understand simple English, Chinese, Malay, or Tamil. Such inclusion criteria were set because we wanted to include participants who had stayed long-term in Singapore, which included both PRs and SCs, and would have the ability to experience and report the differences in lifestyle resulting from the pandemic. With the four main ethnic races being Chinese, Indian, Malay, and Eurasian, the survey was thus only translated to simple English, Chinese, Malay, or Tamil for the majority of the population to understand.

The exclusion criteria established were: (1) individuals who were not SCs or PRs; (2) SCs/PRs who had settled overseas and were not present in Singapore or resided overseas but returned to Singapore from January 2020 to December 2020, as we believed that these individuals would not have lived in Singapore long enough to either experience life before the pandemic and during the pandemic; or (3) individuals who were unable to understand simple English, Chinese, Malay or Tamil, as such individuals would not be able to understand the survey well enough to provide relevant answers and informed consent.

### 2.3. Questionnaire Design

As the study depended on voluntary participation over an online platform, the accuracy and clarity of the survey were critical. Therefore, most of the survey questions were simple, multiple-choice, and closed-ended. The translated questionnaires were back-translated and cross-checked with the English questionnaires, to ensure the preservation of contextual meaning across the different languages. The questionnaire collected participants’ responses to (1) social demographic questions, (2) infection and contact with COVID-19, (3) psychosocial questionnaires assessing hope, optimism, and self-efficacy, and (4) questionnaires evaluating resilience.

#### 2.3.1. Social Demographics and Infection and Contact with COVID-19

Participants were asked for their age, gender, marital status, highest educational qualification, employment status, gross household income, housing type, and the number of individuals living in the same household. Participants were also asked to state whether their friends, family members, or themselves had been infected with COVID-19.

Age, educational qualification, gross household income, and housing type were grouped into categories to ensure equal distribution across all categories. Age brackets were categorized as 21–25 and above 25 years old. Housing type was split into housing and development board (HDB) flats (comprising one- to five-room flats), condominiums, and landed properties. Monthly household income was divided into low (below $6000), middle ($6000–17,500), and high (>$17,500) groups. Education was grouped as secondary education or lower (none, primary school, or secondary school), post-secondary education (junior college, Nitec, or diploma), and university graduates (bachelor’s, master’s, or doctorate).

#### 2.3.2. Psychosocial Questionnaires

The psychosocial questionnaires included the Life Orientation Test (LOT) (10 items), Herth Hope Index (HHI) (12 items), and General Self-Efficacy scale (GSE) (10 items), measuring levels of optimism, hope, and self-efficacy, respectively [16,17].

In the LOT, questions took the form of a 5-point scale, with each response awarding different number of points (1 = strongly agree, 2 = agree, 3 = neutral, 4 = disagree, 5 = strongly disagree) [18]. The sum of items 1, 3, 4, 7, 9, and 10 was indicative of the level of optimism, while items 2, 5, 6, and 10 were filler questions [19]. The points for questions 3, 7, and 9 were reverse-coded prior to scoring. A total score of 0–13, 14–18, and 19–24 correlated with low, moderate, and high optimism, respectively [20].

Participants were also evaluated on various aspects of particularized hope using the HHI [13]. The HHI utilizes a 4-point scale (1 = strongly disagree, 2 = disagree, 3 = agree, 4 = strongly agree), with the scores 12–23, 24–35, and 36–48 reflecting low, moderate, and high levels of hope, respectively [21]. The points for questions 3 and 6 were reverse-coded prior to scoring.

The GSE was used to measure one’s ability to deal with the rapidly changing environment [16]. Participants were questioned on how relatable the statements were to them using a 4-point scale (1 = not true at all, 2 = hardly true, 3 = moderately true, 4 = exactly true) [22].

#### 2.3.3. Measuring Resilience

The Connor-Davidson Resilience Scale (CD-RISC10) was utilized to measure the general level of resilience amongst participants [3]. CD-RISC10 consists of 10 items. The scale utilizes a 5-point scale (0 = not true at all, 1 = rarely true, 2 = sometimes true, 3 = often true, 4 = true nearly all the time) and participants’ scores can range from 0 to 40 [3].

#### 2.3.4. Statistical Analysis

The mean and standard deviation for continuous variables were calculated, as were the numbers and percentages of categorical variables. To determine whether the differences in CD-RISC10 scores between the different sociodemographic categories were statistically significant, *t*-tests and ANOVA analyses were conducted. Linear regression models were constructed to determine the association between sociodemographic factors and CD-RISC10 scores. All factors were added together in the multivariable models to determine their independent association with CD-RISC10 scores. For the psychosocial factors, linear regression models were constructed to determine their association with CD-RISC10 scores. We adjusted these models for age, sex, marital status, and highest educational qualification. The statistical significance was set at *p* < 0.05. All data analysis was conducted using R (version, 4.1.1) (R Foundation for Statistical Computing, Vienna, Austria).

## 3. Results

### 3.1. Study Demographics

Table 1 shows the characteristics of the study sample. The majority of the participants were women (59.2%), were aged between 21 and 25 years (45.5%), and possessed higher education qualifications (degree holders, 52.0%). Most of the participants were also employed (56.2%). A total of 35.2% of respondents reported a monthly household income of less than $6000, with 67.3% living in HDB flats. Table 1 also further summarizes the mean CD-RISC10 scores against individual sociodemographic factors, such as single individuals (25.77 ± 6.25) having a lower mean score than married individuals (27.92 ± 6.34).

### 3.2. Association between Sociodemographic Factors and Resilience

Table 2 shows the pairwise comparisons between sociodemographic factors and CD-RISC10 scores using *t*-tests. Men were found to have higher CD-RISC10 scores compared to women (28.13 vs. 25.54, *p*-value < 0.001). Participants living in landed properties were found to have higher CD-RISC10 scores compared to those living in HDB apartments (28.60 vs. 26.20, *p*-value = 0.033). Married individuals had higher CD-RISC10 scores compared to individuals who were single (25.77 vs. 27.92, *p*-value = 0.0066). Participants who knew of family members or friends infected with COVID-19 had higher CD-RISC10 scores compared to individuals who did not (28.09 vs. 26.19, *p*-value = 0.021).

Table 3 shows multivariate analyses between sociodemographic factors and CD-RISC10 scores where all sociodemographic factors were added together in the model. In general, our results remained consistent, whereby male sex, marital status, and previous encounter or infection with COVD-19 were significantly associated with higher CD-RISC10 scores. These associations were independent of other sociodemographic factors.

### 3.3. Association between Psychosocial Factors and Resilience

Table 4 summarizes the univariate regression analysis of CD-RISC10 scores against psychosocial factors. For the LOT scale, the relevant numbers and percentages were observed as follows: low (50.50%), moderate (43.07%), and high (6.44%) categories. Similarly, for HHI the relevant figures were low (2.72%), medium (50.99%), or high (46.29%). A positive association was observed between psychosocial factors (optimism and hope) and resilience in the univariate analysis.

Table 5 shows the multivariate regression analysis between psychosocial factors and CD-RISC10 scores after adjusting for age, sex, marital status, and education. We found that increasing LOT, GSE, and HHI scores were significantly associated with higher CD-RISC10 scores (*p* < 0.001). Similarly, when LOT and HHT were used as categorical variables, we observed that both the moderate and high groups of LOT and HHT, reflecting high optimism and hope, were associated with higher CD-RISC10 scores (*p* < 0.001) compared to the low groups, reflecting low optimism and hope. These associations were independent of age, sex, marital status, and highest educational qualification.

## 4. Discussion

Our study found that male sex, marital status, previous contact, and infection with COVID-19 were associated with increased resilience. Moreover, increased optimism, self-efficacy, and hope were significantly associated with higher levels of resilience independent of age, sex, marital status, and highest educational qualification.

The results showing men having higher resilience compared to women have been supported by past studies [23]. It has been shown that women work in occupations with higher infection risks, such as in the nursing or teaching industries, and are more likely to have psychological stress and face relatively higher retrenchment rates compared to men [23,24]. Furthermore, women tend to take on more domestic responsibilities as a result of the stay-at-home policies [25]. Thus, the cumulative stress placed on women, both at work and home, increases their general levels of stress, thereby resulting in the inability to cope.

Moreover, our study found that being single adversely affected one’s resilience compared to being married. In contrast to other literature conducted before the pandemic, which found little relationship between marital status and resilience [26], it is reported that being married and having strong familial ties serve as protective factors and facilitate increased resilience during the pandemic [27,28]. This emphasizes that strong familial ties are indispensable for their overall well-being in times of crisis, such as in a pandemic [29].

We also found that individuals with friends or family who had contracted COVID-19 tended to be more resilient than those who did not know of others who contracted the disease, contradicting past literature. For example, a study by Tanoue et al., examining the relationship between having a COVID-19-positive family/friend and psychological distress levels, found that respondents with COVID-19-positive close contacts had higher psychological stress scores than those without [30]. Similar results were also reported in studies conducted in China and Australia [31,32]. We postulate that individuals who knew someone who had recovered from COVID-19 could have a better understanding of the various facets of the disease, thereby becoming more confident in handling the disease should they become infected. Moreover, swift implementation of safe-distancing policies in Singapore and fewer COVID-19-related deaths are less likely to elicit fear and more likely to reinforce resilience [29].

Our study highlighted positive associations between optimism, self-efficacy, and hope with higher levels of resilience. Past literature has suggested that optimism confers effective adaptive management of critical life circumstances, adverse situations, and goals [33]. Optimists adopt a more problem-focused manner of coping and rely on positive reframing, acceptance, and humor in uncontrollable situations, such as the COVID-19 pandemic [34]. Additionally, in difficult times, optimists have a lower tendency to resort to avoidance or escapism [35]. In Singapore’s context, where religion and culture are cornerstones in our society, religious and cultural values might be contributing factors to optimism and resilience [36].

In a 2020 study investigating psychosocial status in families with members possessing special educational needs and disabilities during the COVID-19 pandemic, results showed that participants with higher levels of self-efficacy showed lower levels of anxiety [37]. Similarly, in a study investigating the relationship between psychological well-being and coping mechanisms of care home staff during the COVID-19 lockdown, self-efficacy was found to have direct effects on mental health, reducing symptoms of depression, anxiety, and stress [38]. One reason for these findings is that self-efficacy helps develop self-worth and coping strategies [39]. When facing adversity, self-efficacious individuals also have greater control over their thoughts and, hence, persist in their efforts [40]. This allows for successful adaptation to adversities, therefore increasing resilience.

Lastly, our study showed a significant positive correlation between hope and resilience. Previous studies have shown that hope is associated with a positive adaptation to stress [41,42]. It was found that during the early stages of COVID-19, hopeful individuals had a higher likelihood of increased resilience, preventive behavior, and psychological well-being [42]. They tended to be forward-looking, motivated, and work around obstacles to achieve their goals [42], thereby demonstrating resilience by overcoming stressful situations. Another study conducted during the pandemic described that a hopeful outlook supports positive adaptation to stressors, thereby strengthening one’s resilience [43]. It explained that hope is essential during difficult times to help individuals build coping strategies and move on from adversity [43].

However, there are several limitations to our study that need to be considered. Firstly, being a cross-sectional study, the causality between psychosocial/sociodemographic factors and levels of resilience cannot be ascertained. Secondly, selection bias may be present since the sample was collected via a snowball technique and may not have reached some classes or individuals. Furthermore, we are unable to tabulate the number of adults who had access to this survey, as we did not track the number of people aged 21 years and above who used social networking platforms. It is likely that those who responded to the survey were more likely to be more health conscious. Thirdly, the interpretation of psychosocial scales tends to be subjective and dependent on the present COVID-19 situation. As the survey was conducted during a period where COVID-19 cases were at an all-time high, this may have lowered optimism, self-efficacy, and hope, thus decreasing the resilience of participants. Lastly, though we collected information on several social demographic variables, the effects from the residual confounding effects cannot be eliminated.

### Implications on Policymaking

With the long-drawn nature of the COVID-19 pandemic, an efficient healthcare system is insufficient in protecting the psychological health of the community in the long run. Based on the results of our study, we believe that future interventions and public health policies should aim to improve optimism and instill confidence, hope, and self-efficacy in the community. For example, setting up self-help groups led by professionals allows individuals experiencing similar problems to share openly and build mutual support, improving optimism and self-confidence. Furthermore, self-help kits on COVID-19-specific problems, such as coping with retrenchment and skills upgrading, can be made available, allowing individuals to remain hopeful in times of adversities. Additionally, interventions should place more emphasis on including vulnerable groups, such as single individuals and females. Lastly, primary interventions should be in place to address the root cause of lower resilience amongst these groups, e.g., ensuring more equal gender distribution among occupations and increasing emphasis on daily social interactions.

## 5. Conclusions

In conclusion, this study presented new insights into the relationships between sociodemographic and psychosocial factors and resilience. Sociodemographic factors, such as the male gender, being married, and having friends/family previously having tested positive for COVID-19, alongside psychosocial factors, such as optimism, self-efficacy, and hope, exhibited a positive association with resilience. Thus, interventions that are targeted against these factors are paramount in improving the resilience of the community, ensuring that we are future-ready.

## Figures and Tables

**Table 1:** Characteristics of the Study Population.

Characteristic	Sample Population No. (%)	CD-RISC10 * Score Mean (SD)
Sex		
Male	165 (40.84)	28.13 (6.46)
Female	239 (59.16)	25.54 (6.01)
Age		
21–25	184 (45.54)	26.22 (6.51)
>25	220 (54.46)	26.91 (6.15)
Highest educational qualification		
Secondary and Below	15 (3.71)	23.93 (7.75)
Post-Secondary	179 (44.31)	26.21 (6.44)
University	210 (51.98)	27.11 (6.07)
Employment status		
Employed (Full-time)	213 (52.72)	26.95 (6.14)
Employed (Part-time)	14 (3.47)	26.86 (5.71)
Student	150 (37.13)	26.20 (6.71)
Unemployed	27 (6.68)	25.85 (5.95)
Gross household income per month		
Low (<$6000)	142 (35.15)	25.97 (5.62)
Middle ($6000–10,000)	98 (24.26)	26.23 (5.77)
High (>$10,000)	164 (40.59)	27.35 (7.12)
Housing type		
HDB ^†^	272 (67.33)	26.19 (6.15)
Condominium	81 (20.05)	26.7 (7.15)
Landed	49 (12.13)	28.63 (5.50)
Marital status		
Married	131 (32.43)	27.92 (6.34)
Widowed	4 (0.99)	27.00 (9.09)
Divorced	12 (2.97)	29.67 (3.26)
Single	257 (63.61)	25.77 (6.25)
Number of people living in the same household		
1	11 (2.72)	23.18 (9.16)
2–6	365 (90.35)	26.68 (6.27)
>6	28 (6.93)	26.57 (5.90)
Do you have family members or friends infected with COVID-19 ^‡^?		
Yes	85 (21.04)	28.09 (6.81)
No	319 (78.96)	26.19 (6.13)
Were you infected with COVID-19?		
Yes	3 (0.74)	25.33 (6.43)
No	401 (99.26)	26.6 (6.33)

* 10-item Conner-Davidson Resilience score. ^†^ Housing Development Board flat. ^‡^ Coronavirus.

**Table 2:** Pairwise Comparisons between Sociodemographic Factors and CD-RISC10 Scores.

	Means Comparison	*p*-Value
Sex		<0.001 *†
Male vs. Female	28.13 vs. 25.54	<0.001 *
Housing type		0.045 *†
Landed vs. Condominium	28.60 vs. 26.70	0.20
Landed vs. HDB	28.60 vs. 26.20	0.033 *
HDB vs. Condominium	26.20 vs. 26.70	0.80
Marital status		0.0044 *†
Single vs. Married	25.77 vs. 27.92	0.0066 *
Single vs. Widowed	25.77 vs. 27.00	0.98
Single vs. Divorced	25.77 vs. 29.67	0.13
Divorced vs. Married	29.67 vs. 27.92	0.76
Divorced vs. Widowed	29.67 vs. 27.00	0.86
Widowed vs. Married	27.00 vs. 27.92	0.99
Do you have family members or friends infected with COVID-19?		0.021 *†
Yes vs. No	28.09 vs. 26.19	0.021 *

* Level of significance, *p* < 0.05. † *p*-values are for the overall sociodemographic factors: sex, housing type, marital status, and contact with individuals who have had COVID-19, respectively.

**Table 3:** Multivariate Regression Analyses between Sociodemographic Factors and CD-RISC10 Score.

	Beta Estimate (95% CI)	*p*-Value
Sex		
Male vs. Female	2.78 (1.55, 4.00)	<0.001 *
Age		
21–25 vs. >25	−1.28 (−3.06, 0.50)	0.16
Housing type		
Condominium vs. HDB	0.007 (−1.50, 1.51)	0.99
Landed vs. HDB	1.77 (−0.08, 3.62)	0.061
Marital status		
Married vs. Widowed	−3.79 (−10.23, 2.65)	0.25
Married vs. Divorced	−3.19 (−6.78, 0.40)	0.082
Married vs. Single	2.98 (−1.30, 4.66)	<0.001 *
Highest educational qualification		
Secondary and Below vs. Post-Secondary	−3.08 (−6.63, 0.46)	0.088
University vs. Post-Secondary	1.22 (−0.17, 2.61)	0.086
Do you have family members or friends infected with COVID-19?		
Yes vs. No	1.83 (0.39, 3.27)	0.013 *

Models adjusted for age, gender, marital status, highest educational qualification, and knowing family or friends infected with COVID-19. * Level of significance, *p* < 0.05.

**Table 4:** Summary of the Univariate Regression Analysis of CD-RISC10 Scores against Psychosocial Factors.

Summary of Scores				
	No. (%)	Beta Estimate	95% CI	*p*-Value
Life Orientation Test		0.79	0.64, 0.95	<0.001 *
Low	204 (50.50)			
Moderate	174 (43.07)			
High	26 (6.44)			
General Self-Efficacy Scale		0.93	0.84, 1.03	<0.001 *
Herth Hope Index		0.72	0.64, 0.81	<0.001 *
Low	11 (2.72)			
Medium	206 (50.99)			
High	187 (46.29)			

* Level of significance, *p* < 0.05.

**Table 5:** Multivariate Regression Analysis of CD-RISC10 Scores against Psychosocial Factors.

	Beta Estimate	95% CI	*p*-Value
Life Orientation Test			
Continuous	0.75	0.60, 0.90	<0.001 *
Categorical			
Moderate vs. Low	3.57	2.40, 4.73	<0.001 *
High vs. Low	6.41	4.09, 8.73	<0.001 *
General Self-Efficacy Scale			
Continuous	0.89	0.78, 0.99	<0.001 *
Herth Hope Index			
Continuous	0.71	0.62, 0.80	<0.001 *
Categorical			
Medium vs. Low	9.8	11.79, 18.83	<0.001 *
High vs. Low	14.75	11.55, 19.94	<0.001 *

Models adjusted for age, gender, marital status, and highest educational qualification. * Level of significance, *p* < 0.05.

## Data Availability

Data can be obtained upon reasonable request from the corresponding author.
